# Intensification of marrubiin concentration by optimization of microwave-assisted (low CO_2_ yielding) extraction process for *Marrubium vulgare* using central composite design and antioxidant evaluation

**DOI:** 10.1080/13880209.2017.1297837

**Published:** 2017-03-15

**Authors:** Vineet Mittal, Arun Nanda

**Affiliations:** Department of Pharmaceutical Sciences, Maharshi Dayanand University, Rohtak, India

**Keywords:** High performance thin layer chromatography, conventional, SEM, 2,2-diphenyl-1-picrylhydrazyl, green approach

## Abstract

**Context:** *Marrubium vulgare* Linn (Lamiaceae) was generally extracted by conventional methods with low yield of marrubiin; these processes were not considered environment friendly.

**Objective:** This study extracts the whole plant of *M. vulgare* by microwave assisted extraction (MAE) and optimizes the effect of various extraction parameters on the marrubiin yield by using Central Composite Design (CCD).

**Materials and methods:** The selected medicinal plant was extracted using ethanol: water (1:1) as solvent by MAE. The plant material was also extracted using a Soxhlet and the various extracts were analyzed by HPTLC to quantify the marrubiin concentration.

**Results:** The optimized conditions for the microwave-assisted extraction of selected medicinal plant was microwave power of 539 W, irradiation time of 373 s and solvent to drug ratio, 32 mL per g of the drug. The marrubiin concentration in MAE almost doubled relative to the traditional method (0.69 ± 0.08 to 1.35 ± 0.04%). The IC_50_ for DPPH was reduced to 66.28 ± 0.6 μg/mL as compared to conventional extract (84.14 ± 0.7 μg/mL). The scanning electron micrographs of the treated and untreated drug samples further support the results.

**Discussion and conclusion:** The CCD can be successfully applied to optimize the extraction parameters (MAE) for *M. vulgare*. Moreover, in terms of environmental impact, the MAE technique could be assumed as a ‘Green approach’ because the MAE approach for extraction of plant released only 92.3 g of CO_2_ as compared to 3207.6 g CO_2_ using the Soxhlet method of extraction.

## Introduction

**Figure 1.**
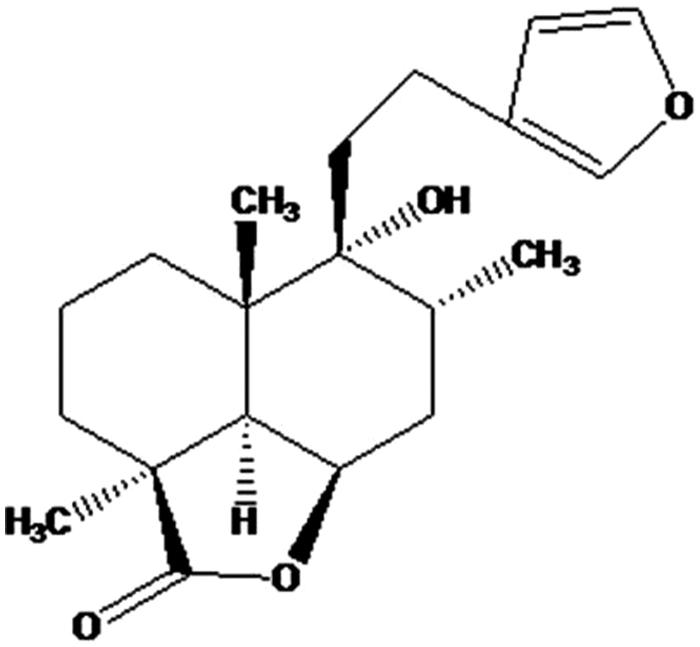
Marrubiin, the chemotaxonomic marker of *Marrubium* genus.

Since ancient times herbs have been the source of a huge range of bioactive compounds such as terpenoids, flavonoids, alkaloids, etc. The safe, effective, and energy efficient extraction of natural products from herbs has always been a challenging task for researchers. The limitations of conventional methods such as high solvent requirement, low quality of extract, huge time consumption, and possible thermal decomposition of target molecules paves the way for the emergence of novel extraction techniques for herbals (Luque de Castro & Garcia-Ayuso 1998). The various techniques have been developed to overcome such limitations like microwave assisted extraction (MAE), ultrasound assisted extraction (UAE), supercritical fluid extraction (SFE), accelerated solvent extraction (ASE), enzyme assisted extraction (EAE), and pressurized liquid extraction (PLE) (Cuoco et al. 2009; Devgun et al. 2009; Gao et al. 2010; Hossain et al. 2011; Mustafa & Turner 2011; Sowbhagya et al. 2011). Moreover, these methods can be assumed as ‘Green approach’ for the extraction of plants. Nowadays the MAE technique is highly acceptable as powerful alternative to traditional methods for the extraction of bioactive compounds from plants. Microwaves are the electromagnetic radiations with a frequency range of 0.3 to 300 GHz. The intensification of extraction process in MAE is mainly attributed to rapid increase in temperature due to high ionic conduction and change in dipole rotations of solvent molecules (Eskilsson & Bjorklund 2000). The heating by microwave is also contributed by the dissipation factor (tan δ) which in turn depends upon the dielectric constant of the material surrounding the solvent (Mandal et al. 2007). Moreover, the moisture inside the plant cell vaporizes due to intense heating and causes the rupturing of cell membrane. The cell disruption facilitates the movement of solvent and solubilization of plant actives thus resulting in high yield of secondary metabolites in relatively less time and with low volume of solvent (Rostagno et al. 2009). The furane labdane diterpene, marrubiin (Figure 1) is assumed as the chemotaxonomic marker among the various species of *Marrubium* genus (Lamiaceae). Generally, marrubiin is isolated from the whole plant of *Marrubium vulgare* Linn which exhibits a vast number of pharmacological properties such as analgesic, antinociceptive, hypotensive, vasorelaxant, antispasmodic, antioxidant, cardioprotective, gastroprotective, antioedematogenic, and antidiabetic activities (De Souza et al. 1998; De Jesus et al. 2000; El Bardai et al. 2001, 2003; Meyre-Silva et al. 2005; Mnonopi et al. 2011; Paula de Olivera et al. 2011; Zaabat et al. 2011; Zhang et al. 2011; Mnonopi et al. 2012). *M. vulgare* is a perennial herb distributed in Kashmir (5000–6000 ft), Europe and North Africa region of world (Kirtikar & Basu 1996). The whole plant is generally extracted by conventional techniques for the isolation of marrubiin (0.3–0.7%) (Rodrigues et al. 1998). For scale-up and industrial purpose of plant actives, the different extraction parameters for medicinal plant could be optimized using various designs. In the present study the central composite design (CCD) coupled with response surface methodology was selected to optimize the extraction conditions for *M. vulgare* using microwaves as heating source. The extracts obtained by Soxhlet method of extraction (SME) and optimized batch of microwave assisted extraction (MAE) were comparatively evaluated for the estimation of marrubiin by high performance thin layer chromatography (HPTLC) and for antioxidant potential. The plant samples were also analyzed by scanning electron micrograph (SEM) for possible microstructural changes.

## Materials and methods

### Plant sample and reagents

The whole plant of *M. vulgare* Linn was collected in the month of June 2013 from the Pulwama district of Jammu and Kashmir state, India. Before processing, the plant was identified by a Dr. Sunita Garg, Chief Scientist, National Institute of Science Communication and Information Resources (NISCAIR) vide reference no NISCAIR/RHMD/Consult/2013/2336-116 dated- 19-11-2013. A voucher specimen was kept in the department for future reference. The marker compound, marrubiin, was purchased from Extrasynthese (France). All the solvents used in the study were of HPLC grade. The ascorbic acid and 2, 2-diphenyl-1-picrylhydrazyl was purchased from Loba Chemie Pvt. Ltd, Mumbai.

### Extraction of plant sample

The selected plant material was dried in shade to remove the moisture and pulverized through a mixer grinder. The powdered sample was sieved to a size of 60–80 mesh before extraction by different methods.

#### Soxhlet method of extraction (SME)

The powdered plant sample (10 g) was extracted with ethanol (500 mL) at 80 °C in a Soxhlet apparatus until complete exhaustion of drug.

#### Microwave-assisted extraction (MAE)

The processed plant sample (5 g) was kept in a four necked round bottom flask with solvent ethanol (50%). The sample was extracted in U-Wave 1000 Microwave synthesis reactor (SINEO Microwave Chemistry Technology, China) at Power-time mode. The instrument operates at an input power of 2000 W with operating frequency of 2450 MHz and works at atmospheric pressure. The real time temperature was monitored by high precision platinum resistance temperature sensor. The flask was connected to outside condenser through a glass connecting tube (19 mm Φ) and a Ч shaped tube. The pulverized drug was extracted at different operating conditions (Microwave power, irradiation time and different volume of solvent/g of drug) as suggested by experimental design. The extracts obtained by different techniques were cooled and centrifuged at 2000 rpm for 5 min before filtration. Further the extract was filtered and concentrated under reduced pressure by a rotary evaporator at 60 °C. The experiment was conducted in triplicate and percentage yield (w/w) was determined using following formula:

$$\text{Percentage Yield }\left(\text{w}/\text{w}\right)\;=\frac{\text{Weight}\;\text{of}\;\text{extract}}{\text{Weight}\;\text{of}\;\text{drug}\;\text{on}\;\text{dried}\;\text{basis}}\text{×100}$$

The extracts were kept in a desiccator before further analysis (Devgun et al. 2013).

### Experimental design

In the present study three process variables like microwave power (X_1_), irradiation time (X_2_) and volume of solvent per g of drug (X_3_) were selected to investigate the effect of these factors on response, i.e. % yield of marrubiin (R) using central composite design (CCD). The optimum conditions for the microwave assisted extraction of selected plant were also determined with the help of response surface methodology (RSM). As described by design, total sixteen experiments were conducted representing eight factorial points, six axial points and two central points for validation and suitability of model. The actual and coded values of independent factors are given in Table 1. A polynomial second order Equation (1) was used to determine the linear (*β_i_*), quadratic (*β_ii_*), and interaction terms (*β_ij_*), for coded independent variable x_i_ and x_j_ where *β_0_* represents the coefficient of interception and *ɛ* is the error (Prakash Maran et al. 2013a).

(1) $$\text{\%}\;\text{Y}\text{i}\text{e}\text{l}\text{d}\;\text{o}\text{f}\;\text{m}\text{a}\text{r}\text{r}\text{u}\text{b}\text{i}\text{i}\text{n}\;\left(\text{R}\right)=\;=\;\beta_{0}\;+\;\sum_{i=1}^{n}\beta_{i}x_{i}+\sum_{i=1}^{n}\beta_{ii}x_{i}^{2}\;+\;\sum_{i\neq j}^{n}\beta_{i}x_{i}x_{j}\;+\;\varepsilon$$

**Table 1.** Coded levels with actual values of different independent factors.

		Coded level				
Independent factors	Unit	−α	−1	0	+1	+α
Microwave power (X_1_)	Watt	164	300	500	700	836
Irradiation time (X_2_)	s	31	150	325	500	619
Solvent to drug ratio (X_3_)	ml/g	3	15	33	50	62

The statistical analysis and significance of proposed model was determined by the application of analysis of variance (ANOVA). The design expert software (7.0.3, Statease Inc, Minneapolis, MN, trial version) was employed for design, analysis and to draw the response surfaces.

### Quantification of marrubiin by HPTLC

A previously developed and validated analytical method, using high performance thin layer chromatography (HPTLC) system (CAMAG, Muttenz, Switzerland), in our laboratory was used for the quantification of marrubiin in various extracts. The extracts were dissolved in methanol (1 mg/mL) and filtered through 0.45 μm membrane filter before analysis. The standard solution of marrubiin was prepared in a concentration of 1 mg/mL and was further diluted to get a final concentration of 10 μg/mL. The precoated plates of silica gel 60 F_254_ (20 × 10 cm) supported on aluminum sheet were used as stationary phase. The ten spots of marker compound was applied (40–400 ng) using CAMAG automatic sample applicator (Linomat V) with the help of micro-syringe (100 μL). The chromatogram was developed using toluene, ethyl acetate, and acetic acid (5:4:1) as mobile phase. The chromatogram was developed by ascending technique up to 80% height of plates. The developed plates were dried at room temperature and heated at 110 °C for 5 min on CAMAG TLC plate heater. The spots were visualized in short UV light and scanned in UV-Visible range using CAMAG TLC densitometric scanner. The WINCATS 1.4.8 software was used to analyze the data. The area under curve (AUC) for different concentration of standard compound (marrubiin) was calculated and standard curve was plotted of AUC versus concentration. The concentration of marrubiin in different extracts was determined using equation of straight line derived from calibration curve (Samaddar et al. 2013; Nanda & Mittal 2016).

### Measurement of DPPH scavenging potential

The antioxidant potential of extracts was measured as capacity to scavenge the free radical of DPPH (2,2-diphenyl-1-picrylhydrazyl). The test solution was prepared by diluting the 2 mL of DPPH solution (0.5 mM in ethanol) with 0.2 mL of extract/ascorbic acid and 2 mL of ethanol. The absorbance of the test solution was measured after 0 min and 30 min of incubation by spectrophotometric method at 517 nm. The % inhibition of extracts has been calculated by using following formula:

$$\text{D}\text{P}\text{P}\text{H}\;\text{I}\text{n}\text{h}\text{i}\text{b}\text{i}\text{t}\text{i}\text{o}\text{n}\;\left(\text{\%}\right)\;=\;\frac{\text{A}{\text{b}}_{\left(\text{t}=0\right)}-\;\text{A}{\text{b}}_{\left(\text{t}=30\right)}}{\text{A}{\text{b}}_{\left(\text{a}\text{t}\;\text{t}=0\right)}}$$

where Ab _(t = 0)_ is the absorbance of test solution before the reaction at 0 min and Ab _(t = 30)_ represents the solution absorbance after 30 min of incubation. The concentration of the solution to scavenge the 50% DPPH (IC_50_) was determined by analysis of dose response graph plotted between the different concentration of test solution (20–40 μg of sample/mL of ethanol) and inhibition (%). All the experiments were carried out in triplicate and results were expressed as mean ± S.D. The significance of results was determined by ANOVA followed by Tukey test (Bersuder et al. 1998).

### Scanning electron microscopy (SEM)

After extraction with different methods, any change in the microstructure of drug sample was confirmed by SEM analysis. The micrographs of treated sample were compared with the untreated plant sample. The samples were subjected to thermal treatment at 40°–50 °C under vacuum for 2 h, sputter coated and then examined with scanning electron microscope (Zhou & Liu 2006).

## Results and discussion

The whole plant of *M. vulgare* was extracted by the Soxhlet method of extraction (SME) and the yield of extract was 11.27 ± 1.45% (w/w). The linear regression equation for calibration curve was *Y* = 4.132 *X* + 13.02; where *Y* was area under curve (AUC) and *X* stands for the concentration of marrubiin (μg). The significant value (0.99) of coefficient of determination (*R*^2^) suggests that the equation can be used to evaluate the concentration of marrubiin in different extracts. The percentage yield (%) of marrubiin in the extract obtained by SME was 0.69 ± 0.08.

**Figure 2.**
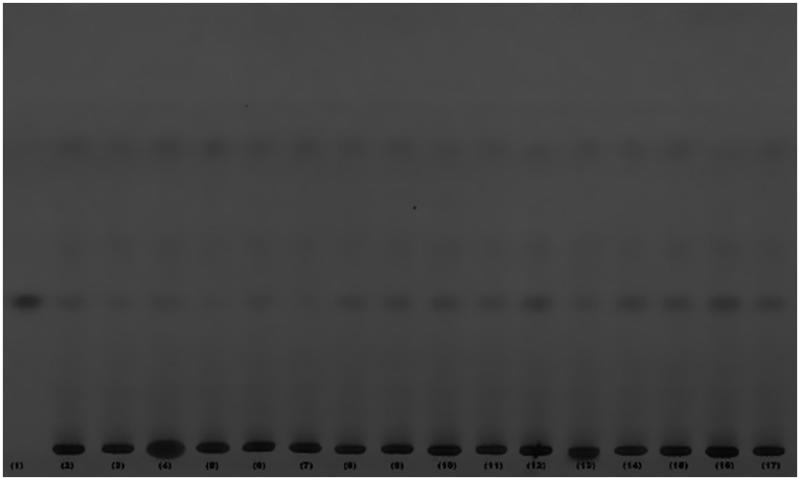
TLC chromatogram of different extracts of MAE obtained in 16 experiments (2–17) along with pure marrubiin (1) at 254 nm (UV light).

**Figure 3.**
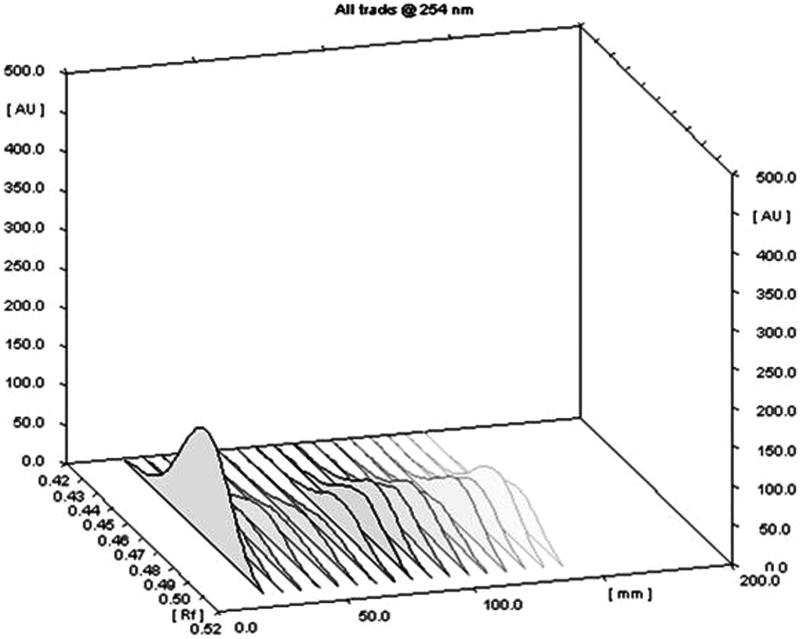
3 D diagram showing AUC for marrubiin in different extracts of MAE.

Generally, a variety of factors influence the productivity of active constituents by MAE process. It includes the particle size, microwave power, irradiation time, extraction temperature, nature of solvent, solvent concentration, solvent to feed ratio, etc. In the present study the ethanol was used as solvent as it is a nontoxic and universal solvent. Also the mechanism of MAE suggested that the generation of heat during extraction also depends upon the dielectric constant of solvent. The solvent absorbs the radiations and dissipates it in the form of heat. The dissipation factor is directly proportional to dielectric constant of the solvent therefore the solvent with high dielectric constant, like water (near 78 at about 80 °C) and alcohol (near 25 at about 70 °C) can be mixed to have a solution which can be used for the efficient extraction in MAE. Moreover, the ethanol is a good absorber of microwaves and is generally considered the best for microwave based extraction of active constituents from herbs (Zhou & Liu 2006). Keeping in mind the stability, yield of active constituent (marrubiin) and physical properties of solvent, the temperature for extraction was kept at 80 °C (Chan et al. 2011). Also on the basis of some of our preliminary experiments the concentration of solvent was selected as 50% for all the extraction procedure by MAE. Many researchers had applied the different designs for the optimization of MAE process for herbs (Zheng et al. 2009). In the present study the response surface methodology (RSM) using central composite design (CCD) was successfully employed for optimization of various extraction parameters in MAE for *M. vulgare*. A total of 16 experiments were conducted to study the effect of three selected independent factors (X_1_, X_2_ and X_3_) on the % yield of marrubiin (R). A chromatogram of all the sixteen extracts and pure marrubiin was simultaneously developed to confirm the presence of marrubiin (R_f_ 0.48) in each extract (Figure 2). The developed plates were scanned by CAMAG TLC densitometric scanner and 3 D diagram showing area under curve (AUC) corresponding with same R_f_ (0.48) was considered for the quantitative estimation of marrubiin in each extract (Figure 3). The marrubiin yield (%) for various experimental conditions as prescribed by CCD was calculated by straight line equation (Table 2).

**Table 2.** % Yield of marrubiin (*R*) under various experimental conditions as prescribed by CCD.

	Actual values of different variables				% Marrubiin Yield (*R*)	
Run	X_1_	X_2_	X_3_	Extract Yield (%)	Actual	Predicted
1	700 (+1)	150 (−1)	15 (−1)	15.16	0.88	0.93
2	164 (−α)	325 (0)	33 (0)	12.04	0.75	0.81
3	700 (+1)	150 (−1)	50 (+1)	14.1	0.80	0.79
4	300 (−1)	150 (−1)	15 (−1)	11.3	0.684	0.683
5	300 (−1)	150 (−1)	50 (+1)	11.5	0.69	0.61
6	500 (0)	31 (−α)	33 (0)	8.3	0.56	0.61
7	500 (0)	325 (0)	3 (−α)	14.64	0.85	0.88
8	500 (0)	619 (+α)	33 (0)	17.96	1.09	1.14
9	700 (+1)	500 (+1)	15 (−1)	18.18	1.14	1.14
10	300 (−1)	500 (+1)	50 (+1)	16.24	0.92	0.91
11	836 (+α)	325 (0)	33 (0)	18.04	1.12	1.16
12	500 (0)	325 (0)	62 (+α)	12.62	0.78	0.84
13	500 (0)	325 (0)	33 (0)	19.84	1.32	1.32
14	700 (+1)	500 (+1)	50 (+1)	19.78	1.24	1.17
15	500 (0)	325 (0)	33 (0)	20.48	1.34	1.32
16	300 (−1)	500 (+1)	15 (−1)	16.34	0.99	0.93

Values in bracket show the coded level of the independent variable.

The design expert software was used to apply the analysis of variance (ANOVA) to experimental data to find out statistical significance of the model (Table 3). The model exhibits the *F* value of 16.13 which implies that there was only 0.15% chance that such large *F* value occurs due to noise. Moreover, the lower *p* value (<0.001) further confirms that the developed model was highly significant and regression equation can explain the difference in response. The coefficient of determination (*R*^2^) measures the degree of fitness for model and is the ratio of explained variation to total variation. In present case, the *R*^2^ approaches to unity (0.96) which suggests the better fitness of model with the actual data and only 3.4% of total variations are not explained by this model (Nath & Chattopadhyay 2007). In addition, the adjusted *R*^2^ (0.90) was comparable to *R*^2^ which further explains that the selected form of developed model used to establish the relationship between the independent variables and response was very well correlated. Further the lack of fit for the developed model has *p* value <0.129 and *F* value of 33.87 which indicates that it was not significant relative to the pure error. The small value of coefficient of variance (7.96) clearly explains the reliability and precision of conducted experiments signal to noise ratio of 11.97 justifies the fitness of model to navigate the design space (Dahmcune et al. 2015).

**Table 3.** Analysis of variance (ANOVA) for experimental data and estimated coefficient for various variables.

Parameter	Estimated coefficient	Sum of square	df	*F* value	*p*-value Prob>*F*
Model intercept	1.32	0.82	9	16.13	0.001**
Linear					
X_1_	0.10	0.14	1	25.32	0.002**
X_2_	0.16	0.33	1	58.52	0.0003***
X_3_	0.012	1.98	1	0.35	0.57
Interaction					
X_1_ X_2_	0.020	3.24	1	0.57	0.47
X_2_ X_3_	0.013	9.46	1	0.17	0.69
X_1_ X_3_	0.011	1.43	1	0.25	0.63
Quadratic					
X_1_^2^	−0.12	0.13	1	23.17	0.003**
X_2_^2^	−0.16	0.23	1	40.76	0.0007***
X_3_^2^	−0.16	0.24	1	42.60	0.0006***
Lack of Fit		0.034	5	33.87	0.129
R^2^	0.96				
Adjusted *R*^2^	0.90				
C.V% (coefficient of variance)	7.96				

df: degree of freedom.

### Model fitting

A mathematical model was developed by multiple regression analysis of experimental data. The model represents the coefficient for various variables and was also used to study the relationship between the response and independent variables. The second order polynomial Equation (2) developed in terms of coded variables is as follows:

(2) $$R=\text{ 1}.\text{32}+\;0.\text{1}0{\text{ X}}_{\text{1}}+\;0.{\text{16 X}}_{\text{2}}-\;0.0{\text{12 X}}_{\text{3}}+\;0.0\text{2}0{\text{ X}}_{\text{1}}{\text{X}}_{\text{2}}+\;0.0{\text{11 X}}_{\text{1}}{\text{X}}_{\text{3}}+\;0.0{\text{13 X}}_{\text{2}}{\text{X}}_{\text{3}}-\;0.{\text{12 X}}_{\text{12}}-\;0.{\text{16 X}}_{\text{22}}-\;0.{\text{16 X}}_{\text{32}}$$

The coefficient of linear terms (X_1_ and X_2_) and all the quadratic terms were highly significant (*p* < 0.01) whereas the interactive term coefficients and one of the linear term coefficient (X_3_) were not considered to be significant on the basis of *p* value (*p* > 0.05) (Table 3).

### Diagnostics of model adequacy

**Figure 4.**
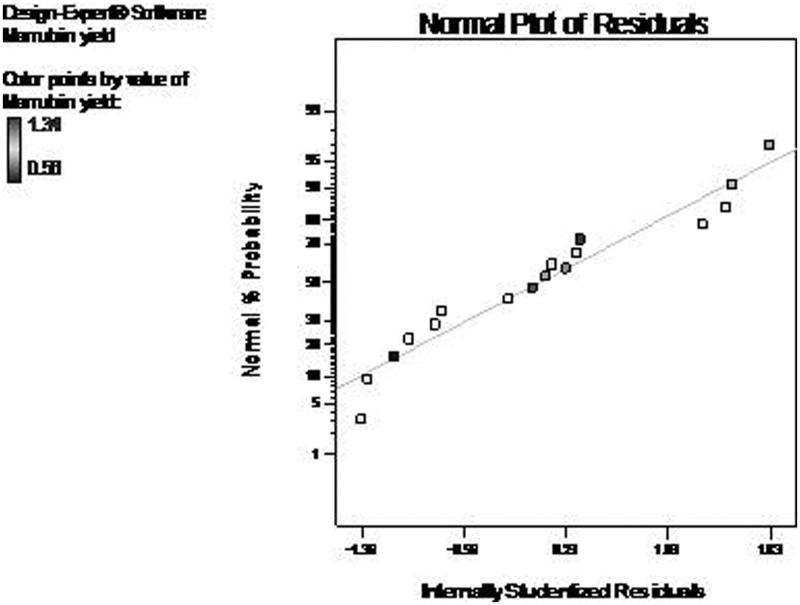
Normal plot of residual between normal % Probability and internally studentized residuals.

**Figure 5.**
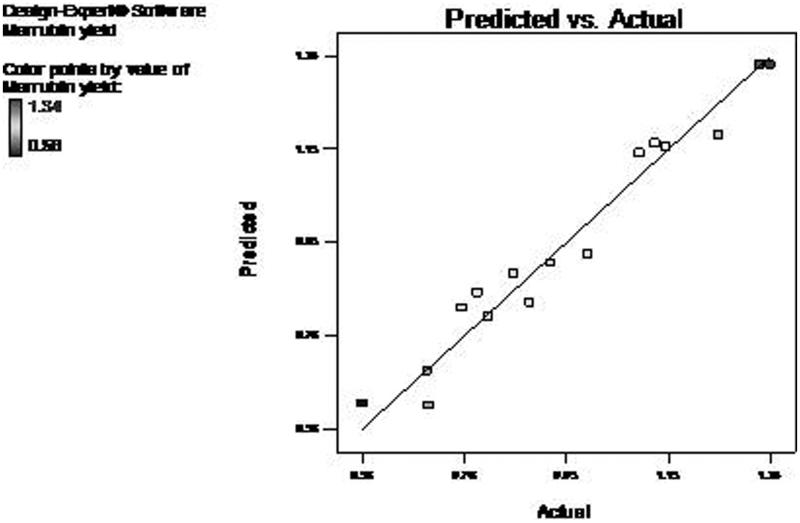
Plot between predicted vs. actual response.

The adequacy of developed model was further evaluated by diagnostic plots. The normal plot of residual between normal % probability and internally studentized residuals was normally distributed with no significant deviation of variance which justifies the fitness of developed model (Figure 4). The plot between predicted and actual response clearly indicated that all the predicted values lie near to the straight line and was in agreement with real values (Figure 5) (Thirugnanasambandham et al. 2015).

### Response surface analysis

**Figure 6.**
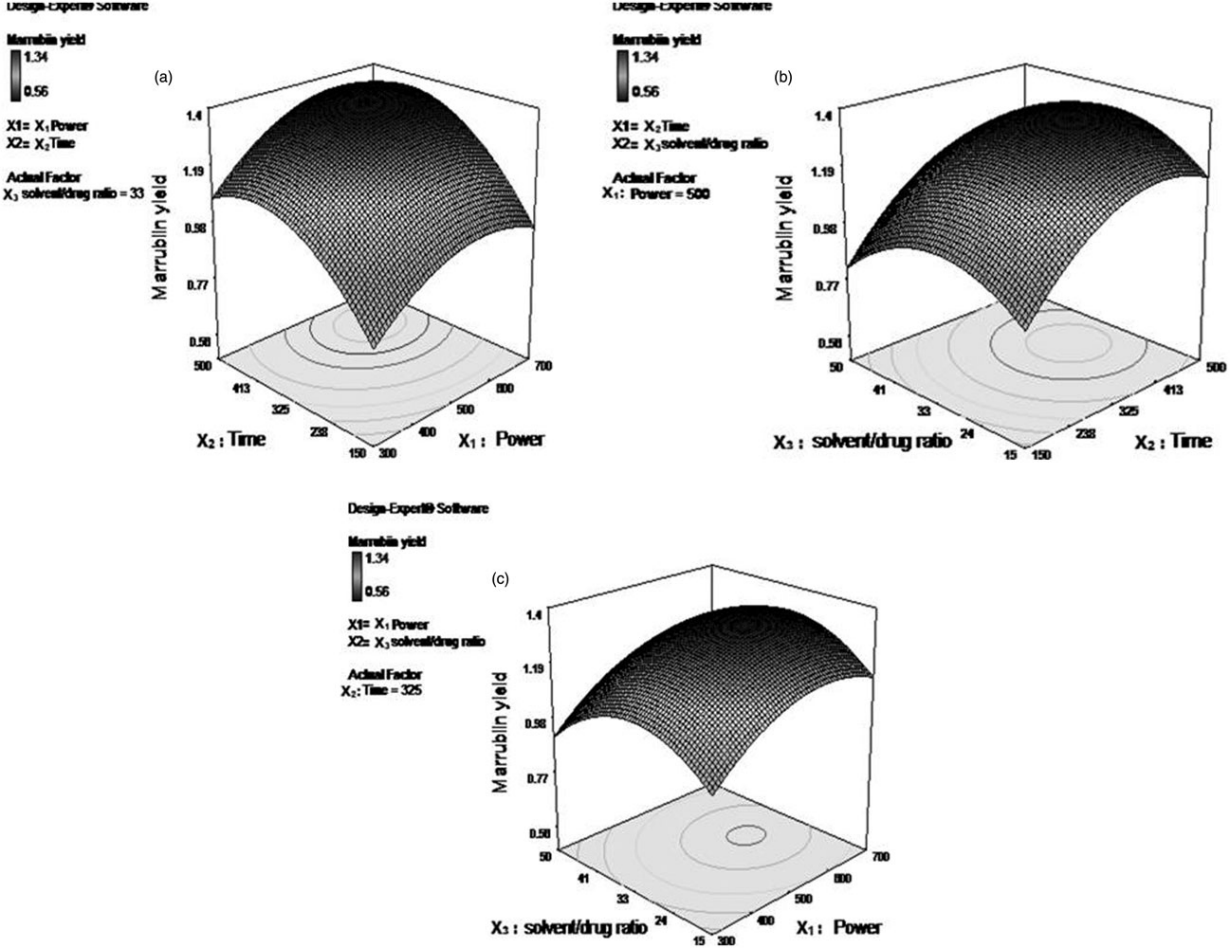
Response surface analysis for % marrubiin yield (*R*) from *Marrubium vulgare* L. by microwave assisted extraction with respect to (a) X_1_ (microwave power) and X_2_ (irradiation time). (b) X_2_ (irradiation time) and X_3_ (solvent to drug ratio). (c) X_1_ (microwave power) and X_3_ (solvent to drug ratio).

The 3 D response surface graph predicts the relationship between the response and two process variables, keeping the third factor at zero level. The effect of microwave power (X_1_) and irradiation time (X_2_) on percentage yield of marrubiin (R) was shown in Figure 6(a) while the solvent/drug ratio (X_3_) was kept at zero level. It was clear from the figure that the yield of marker compound increases linearly with power up to 500 W, after that rise in response was not so significant up to 620 W and further increases in power slightly decrease the marrubiin yield (%). It could be explained as the power rises with time more it would more dissipated as heat inside the plant cell and extractant. The intensity of heat depends mainly on dipole rotation and ionic conduction of molecules of dispersion medium. The dipoles of the solvent molecules undergo oscillation and get aligned in the direction of electric field. This process of oscillation and alignment of dipoles occurs at a very high rate which generates the heat inside the medium (Eskilsson & Bjorklund 2000). Moreover, the solvent molecules also get dissociated into charged particles (ions) and their flow inside the vessel was also enhanced under the effect of applied electric field. The increased movement of ions also causes the enhanced collision of charged particles and generation of friction energy which ultimately rise the temperature of the medium (Gfrerer & Lankmayr 2005; Nadagouda et al. 2011). This heat also vaporizes the moisture present inside the drug particles and the thermal effect of microwaves inside the cells of the drug particles causes the cracks or fracture on the cell wall of plant matrix which in turn results in enhanced penetration of solvent inside the cell and more solubilization and exudation of target analytes in the surrounding liquid occurs (Rostagno et al. 2009).

After the complete exhaustion of plant cells, the microwave power did not show any significant effect on concentration of active constituents (Alfaro et al. 2003). Still, if the herb was exposed to higher power it would deteriorate the chemical structure of active compound to cause decrease in its yield (Hao et al. 2002). The exposure time to microwaves also plays a crucial role in the extraction of chemical constituents from botanicals. From the response surface plot of irradiation time (X_2_) and solvent to drug ratio (X_3_) at a fixed power level (500 W), it was observed that the concentration of marker compound increased steadily up to 369 s (Figure 6(b)). This type of behaviour could be attributed to the higher thermal accumulation of solvent due to absorption of electromagnetic radiation with time. However, over exposure of plant material to radiation may decompose the phytoconstituents and finally results in lower efficiency (Wang et al. 2009). The influence of solvent volume relative to drug sample (solvent per g of drug) was also evaluated. The 3 D plot between the irradiation power (X_1_) and solvent per g of drug (X_3_) for 325 sec (mid level) established the relationship independent variable (X_3_) and dependent variable (*R*) (Figure 6(c)). The observation of plot reveals the linear rise of marrubiin yield with solvent concentration upto 30 mL/g of drug, because the optimum amount of solvent breaks the mass transfer barrier and promotes the movement of plant actives out of the cell matrix (Prakash Maran et al. 2013b).

Further, the large solvent to feed ratio results in slight decrease in the yield of marrubiin from the extract. This observation was possibly due to fact that the large volume of solvent requires the additional power for effective heating which in turn disturbs the chemical nature of constituents. Moreover, at higher ratio the mass transfer rate was negatively affected and it stops the inoculation of active constituent (marrubiin) in solution and thus decreases its yield (Mandal & Mandal 2010).

### Optimization of extraction parameters and validation of predictive model

The research protocol aims for the optimization of selected extraction parameters to get maximum yield of marrubiin. In the design expert software, the numerical optimization approach evaluates the experimental data and X_1_ of 539 W, X_2_ of 373 seconds and X_3_ of 32 mL per g of drug was predicted as optimized conditions to get the maximum yield of marrubiin (1.368%) which was not significantly different from predicted response and thus validates the model.

### Comparative analysis of MAE with SME

**Figure 7.**
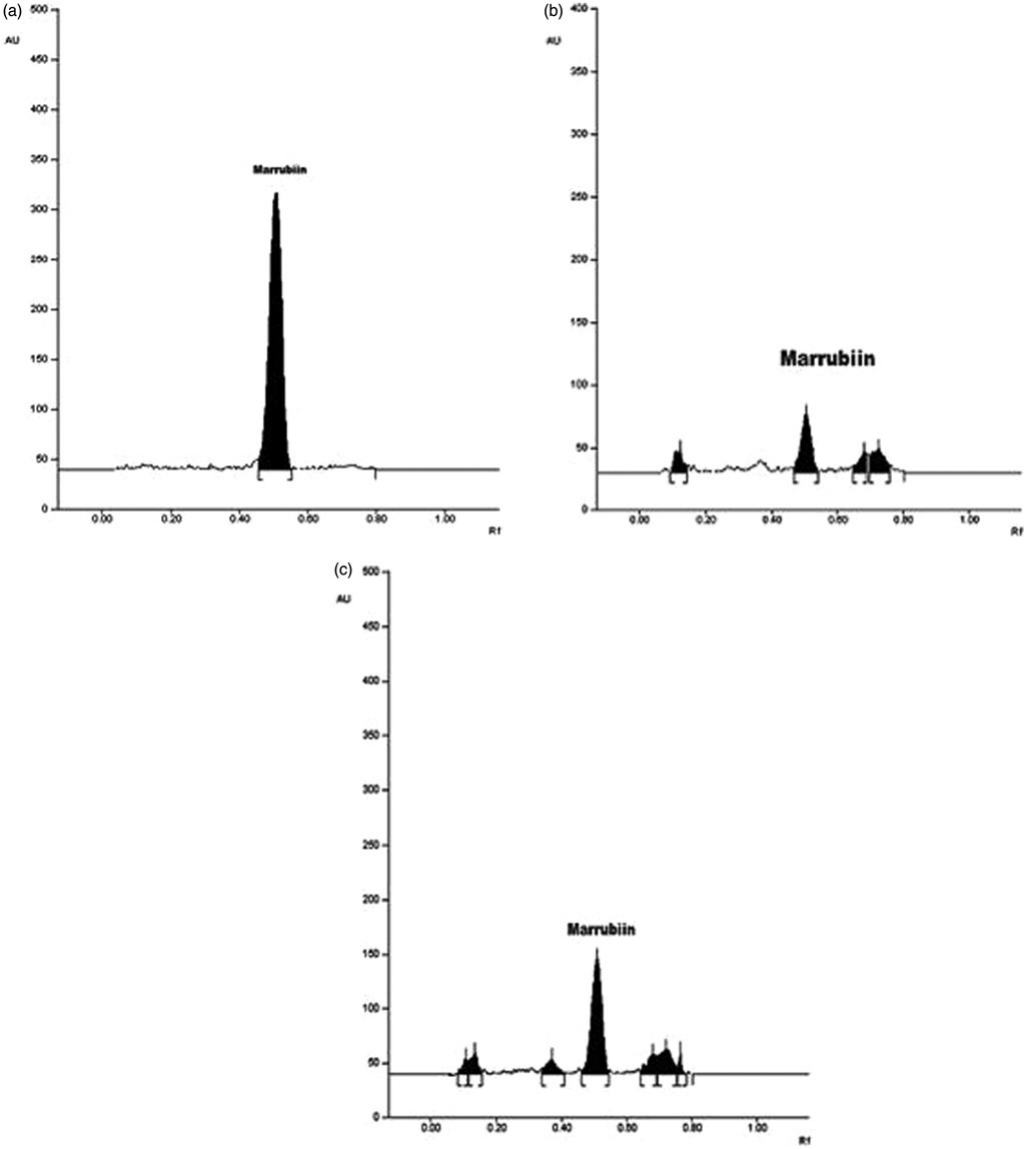
(a). Chromatogram showing AUC for the marrubiin. (b) Chromatogram showing AUC for the conventional extract (SME). (c) Chromatogram showing AUC for the extract obtained by MAE.

The microwave-assisted extraction (at optimized conditions) and SME was carried out in triplicate and marrubiin concentration was quantified by HPTLC as described in previous section. The chromatogram of pure marrubiin indicated that the retention factor for the compound was 0.48 (Figure 7(a)). The TLC chromatogram of SME (Figure 7(b)) and MAE (Figure 7(c)) confirms that the marrubiin was present in both extracts and the yield was significantly increased from 0.69 ± 0.08 (%) in conventional extract to 1.35 ± 0.04 (%) in MAE.

#### Radical scavenging activity

**Figure 8.**
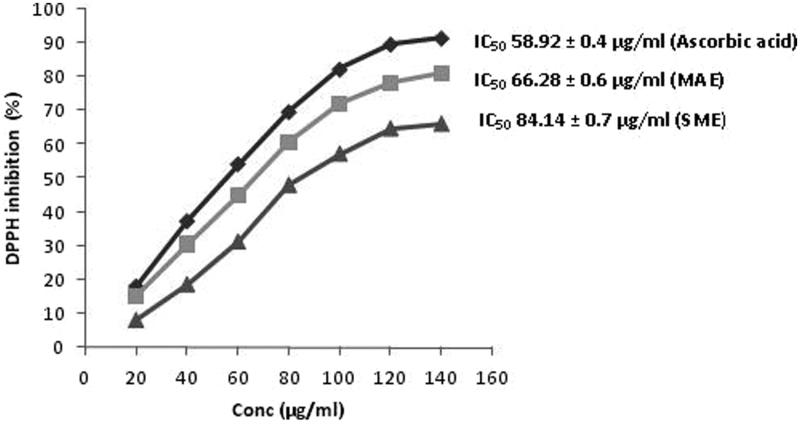
Concentration-based DPPH inhibition (%) of various extracts and ascorbic acid (standard).

The DPPH solution accepts the hydrogen atom from substrate present in the extract and gets reduced. The violet colour of DPPH solution gets faded due to reducing capability of substrate in extract (Ardestani & Yazdanparast 2007). The concentration-based quenching of DPPH radical by test samples (Figure 8) indicates that the MAE extract significantly reduced the IC_50_ to 66.28 ± 0.6 μg/mL relative to the 84.14 ± 0.7 μg/mL of the extract obtained by conventional method. The reduced DPPH scavenging potential of the microwave extract could be attributed to increased quality of extract in terms of marrubiin concentration as well as the phenolic and flavonoids compounds.

#### Microstructural analysis by SEM

**Figure 9.**
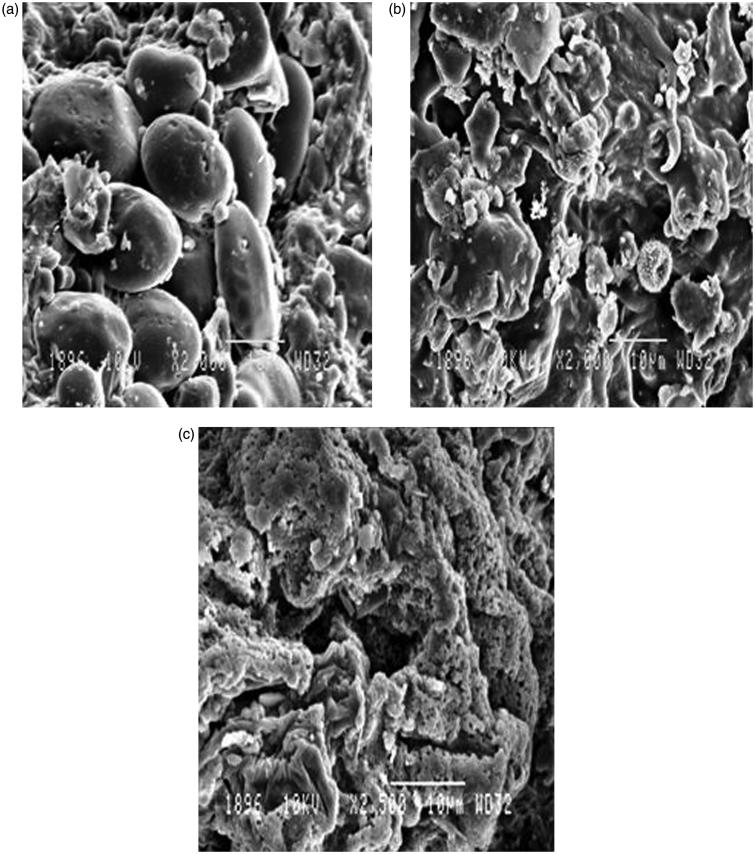
(a) SEM image of drug particles before extraction. (b) SEM image of drug particles after extraction with SME. (c) SEM image of drug particles after extraction by MAE.

The micrographs of the untreated sample indicated the consistent and uniform cellular structure of drug particles (Figure 9(a)) whereas some disorganized cells was observed after extraction with Soxhlet method (Figure 9(b)). The rapid rise in temperature and internal pressure by MAE process crumbled the uniformity and texture of vegetal cells significantly (Figure 9(c)). This situation augments the inlet movement of solvent, solubilization of components and exudation of constituents from cell wall causing enhancement of process efficiency (Ferhat et al. 2006; Zhang et al. 2008).

#### Impact on environment and energy consumption

The MAE process in optimal conditions (at 539 W and for 373 s) significantly reduced the cost of extraction as compared to SME (at 200 W for 18 h). The energy consumption was cut down from 3.6 KWH in conventional method to 0.103 KWH by MAE process. In terms of environmental impact, the selected novel technique of extraction (MAE) could be assumed as ‘Green approach’ for extraction of whole plant of *M. vulgare*. As this process release only 92.30 g of CO_2_ relative to 3207.6 g of carbon dioxide in traditional method (SME) (Filly et al. 2014; Abdelhadi et al. 2015). Further, in the present study the solvent ratio per g of the drug was also optimized to 32:1 which suggest the less consumption of solvent in MAE process in comparison to the Soxhlet method of extraction (50:1). The comparative data of various extracts is summarized in Table 4.

**Table 4.** Comparison of different extracts for extract yield (%), marrubiin concentration (%), IC_50_ and CO_2_ emission.

Extraction technique	Power (Watt)	Time	Solvent to drug ratio (mL/g of drug)	Yield of extract (%)	Marrubiin concentration (%)	IC_50_ for DPPH inhibition (μg/ml)	CO_2_ emission (g)
MAE	539	373 (s)	32	20.48 ± 1.01**	1.35 ± 0.04**	66.28 ± 0.6**	92.3
SME	200	18 (h)	50	11.27 ± 1.45	0.69 ± 0.08	84.14 ± 0.7	3207.6

** *p* value <0.01 and is considered significant.

## Conclusion

The present research work concludes that the central composite design coupled with response surface methodology can be successfully applied to optimize the extraction parameters (MAE) for *M. vulgare*. The study reveals that extraction of selected medicinal plant by microwave based approach at optimized conditions almost doubles the extract yield and marrubiin concentration with less consumption of solvent and electricity as compared to traditional method. Moreover, the DPPH scavenging potential of the MAE extract enhanced significantly relative to the SME. The enhanced anti-oxidant activity may also be due to altered concentration of phenolic or flavonoid compounds which can be further evaluated. The results were also supported by SEM analysis of drug samples. To conclude, it can be said that MAE technique for extraction of whole plant of *M. vulgare* could be commercially utilized for scale-up process. Further MAE could be assumed as green approach for the extraction with high returns on capital investment. Finally the research could be further extended to analysis of extracts for other phytoconstituents and also the variability of contents with different collection time of raw material.
